# Characterization of cyclophilin D in freshwater pearl mussel (*Hyriopsis schlegelii*)

**DOI:** 10.24272/j.issn.2095-8137.2017.018

**Published:** 2017-03-18

**Authors:** Xiu-Xiu Liu, Cheng-Yuan Wang, Chun Luo, Jun-Qing Sheng, Di Wu, Bei-Juan Hu, Jun-Hua Wang, Yi-Jiang Hong

**Affiliations:** ^1^School of Life Sciences, Nanchang University, Nanchang Jiangxi 330031, China; ^2^Key Laboratory of Aquatic Animals Resources and Utilization of Jiangxi, Nanchang University, Nanchang Jiangxi 330031, China

**Keywords:** *Hyriopsis schlegelii*, Cyclophilin D, Sequence analysis

## Abstract

Cyclophilin D (referred to as *HsCypD*) was obtained from the freshwater pearl mussel (*Hyriopsis schlegelii*). The full-length cDNA was 2 671 bp, encoding a protein consisting of 367 amino acids. HsCypD was determined to be a hydrophilic intracellular protein with 10 phosphorylation sites and four tetratricopeptide repeat (TPR) domains, but no signal peptide. The core sequence region YKGCIFHRIIKDFMVQGG is highly conserved in vertebrates and invertebrates. Phylogenetic tree analysis indicated that CypD from all species had a common origin, and HsCypD had the closest phylogenetic relationship with CypD from Lottia gigantea. The constitutive mRNA expression levels of *HsCypD* exhibited tissue-specific patterns, with the highest level detected in the intestines, followed by the gonads, and the lowest expression found in the hemocytes.

## INTRODUCTION

Cyclophilin is a type of intracellular receptor of cyclosporin A (CsA). It has a peptidyl-prolyl cis-trans isomerase (PPIase) region and can be combined with CsA (Feng & Xin, 2013). Widely found *in vivo*, cyclophilins have a conserved structure and biological function. Cyclophilin D (CypD) is a mitochondrial matrix protein, and plays a crucial role in protein folding, cell apoptosis, necrosis, and immunosuppression (Thomas et al., 2012). The CypD protein protect cells from death that is induced by oxidative stress and mediated by mitochondria (Basso et al., 2005). It is a key factor in the regulation of the mitochondrial permeability transition pore (MPTP), which plays a role in the release of cytochrome C and other apoptotic factors from mitochondria during cell apoptosis (Forte & Bernardi, 2006). CypD may also interact with mitochondrial adenine nucleotide transporters (ANT, Halestrap et al., 1998; Hunter & Haworth, 1979) and promote "open" conformation of ANT (Hunter & Haworth, 1979). Moreover, CypD can suppress apoptosis when it is overexpressed (Li et al., 2004; Lin & Lechleiter, 2002; Schubert & Grimm, 2004).

Cyclophilins from the freshwater pearl mussel (*Hyriopsis schlegelii*) (Wang et al., 2016) are related to cell growth and immunity (Luo et al., 2015; Xie et al., 2011). However, whether CypD from *Hyriopsis schlegelii* (HsCypD) has a conserved structure remains unclear and its predicted function has yet to be reported. Here, we describe the predicted cDNA sequence and protein structural features of HsCypD.

## MATERIALS AND METHODS

### Experimental animals

Healthy four-year-old *H. schlegelii* individuals (*n*=15), with shell lengths averaging 150.0±10.4 mm, were obtained from the Fuzhou Hongmen Reservoir Exploitation Corporation, Jiangxi Province. They were kept in aerated freshwater at 23±2 ℃ for one week before the tissues were harvested.

### Total RNA extraction, cDNA synthesis, and cloning

Total RNA extraction was performed using TRIzol Reagent (Invitrogen) per the manufacturer's protocols. After the evaluation of RNA quantity, purity, and integrity, RNA from the gonads was used to prepare cDNA with the SMART RACE Kit (Clontech, USA). A cDNA library for *H. schlegelii* was constructed using the SMART cDNA Library Construction Kit (Clontech, USA). The full-length cDNA of *HsCypD* was cloned by RACE methods, with gene specific primers (Supplementary Table 1) based on the known EST sequence.

### Bioinformatics analysis

The cDNA fragments of *HsCypD* were assembled into complete full-length cDNA. The open reading frame was examined using ORF Finder.

Protein molecular weight, isoelectric point (pI), and amino acid composition were analyzed with the Compute pI/Mw function of the ExPASy-ProtParam tool (http://web.expasy.org/protparam/). Protein hydrophobicity was analyzed using ExPASy-ProtScale (http://web.expasy.org/protscale/). Protein subcellular localization was predicted with PSORT Ⅱ (http://www.genscript.com/tools/psort). Signal peptides were predicted using SignalP 3.0 (http://www.cbs.dtu.dk/services/ SignalP-3.0/). The protein folding model was predicted using Predict Protein from Columbia University (https://www.predictprotein.org/). The protein domains were predicted using the Conserved Domain Database (https://www.ncbi.nlm.nih.gov/Structure/cdd/wrpsb.cgi). The three-dimensional structures of the protein sequences were predicted using the ExPASy SWISS-MODEL program (http://swissmodel.expasy.org/).

Alignment of the amino acid sequences of HsCypD, HsCypC, and HsCypH with other species was performed using ClustalW Multiple Alignment (http://www.ebi.ac.uk/clustalw/). A phylogenetic tree was constructed by neighbor-joining (NJ) using MEGA 4.0 (Tamura et al., 2007) with 1 000 bootstrap replicates, based on amino acids alignment.

### mRNA expression analysis by quantitative real-time PCR

Real-time PCR was applied to examine the mRNA levels of *HsCypD* in 10 tissues using primer pairs *CypD*-F and *CypD*-R (Supplementary Table 1). Each assay was performed in triplicate with β-actin as the internal reference. Real-time PCR was performed for each cDNA sample on a Mastercycle ep Realplex2 Real-Time Thermal Cycler (Eppendorf) with SYBR Premix Ex TaqTM (TaKaRa). The 2^-∆∆CT^ method (Schmittgen & Livak, 2008) was used to analyze expression levels. All data were relative mRNAs expressed as mean±*SD* (*n*=3), and subjected to Student's *t*-test.

## RESULTS

### cDNA sequence of *HsCypD* and predicted protein features

**Figure 1:**
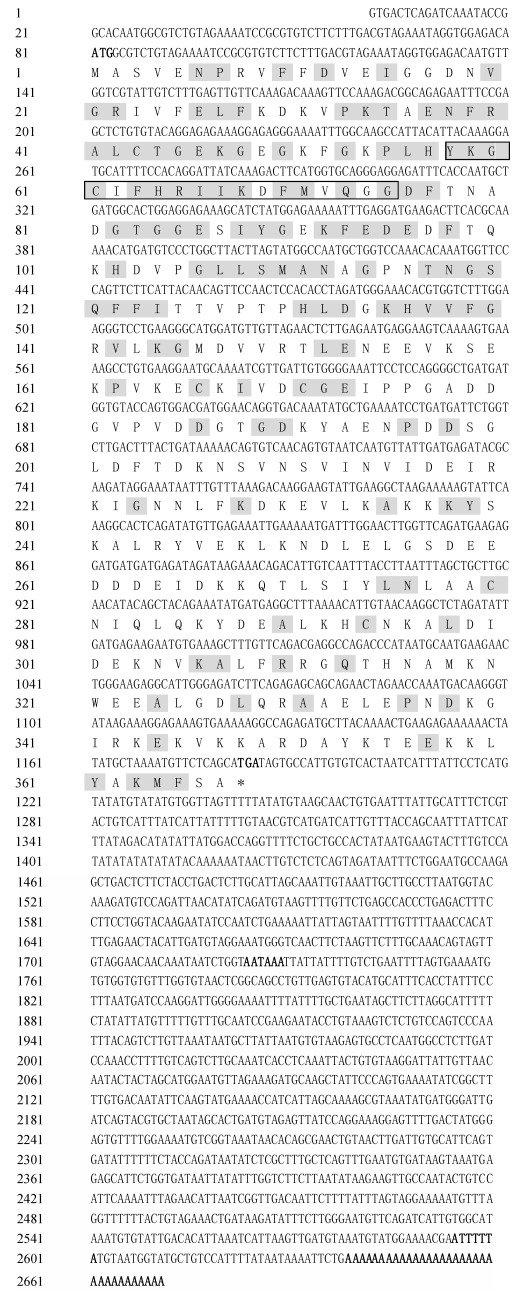
cDNA and deduced amino acid sequence of HsCypD

The complete cDNA sequence of *HsCypD* was 2 671 bp in length, containing a 5'-untranslated region (UTR, 80 bp), 3'-UTR (1 487 bp), polyadenylation signal (AATAAA), and unstable signal (ATTTTTA). The open reading frame (ORF) was 1 104 bp, encoding 367 amino acids (Figure 1). The sequence was deposited in GenBank under accession number KJ747387. The predicted protein of *HsCypD* had a predicted isoelectric point (pI) of 5.43. The largest portion of the HsCypD residues was hydrophobic (131 amino acids), followed by uncharged polar amino acids (103 amino acids) (Supplementary Table 2). The hydrophobicity score of HsCypD was highest (1.544) at the 24^th^ and 275^th^ sites and lowest (-2.856) at the 260^th^ site (Supplementary Figure 1). Hydrophobicity of HsCypD was not obvious on the atlas, but the strength of hydrophilicity was clear. From the 330^th^ to 360^th^ residues, there was a small area of hydrophilicity that appeared to be very dense (Supplementary Figure 1). Moreover, the grand average of hydropathicity (GRAVY) of HsCypD was -0.703, thus showing obvious hydrophilicity. The proportion of hydrophilic amino acids (about 64%) was much larger than that of hydrophobic amino acids (about 36%) (Supplementary Table 2). Therefore, HsCypD was predicted to be a hydrophilic protein.

Protein subcellular localization results showed that 69.6%, 8.7%, and 4.3% of the HsCypD protein was distributed in the cytoplasm, cell nucleus, and cell membrane, respectively. The distribution of the protein inside the cell was higher than that outside, indicating an intracellular protein. No obvious signal peptide characteristics were observed at the N-terminal (Supplementary Figure 2A, B).

Eight possible folding patterns of HsCypD were identified: a N-glycosylation site at 118-122; casein kinase Ⅱ (CKII) phosphorylation sites at 78-82, 83-87, 199-203, and 257-261; N-myristoylation sites at 74-80, 177-183, 200-206, and 312-318; protein kinase C (PKC) phosphorylation sites at 99-102, 159-162, and 204-207; a cyclophilin peptidyl-proline cis-trans isomerase signal area at 58-76; CAMP and CGMP dependent protein kinase phosphorylation sites at 237-241 and 267-271; a leucine zipper model at 277-299; and a tyrosine kinase phosphorylation site at 354-362 (Supplementary Table 3). The secondary structures observed not only included α-helices, but also β-pleated sheets, β-turns, and random coils. Among them, α-helices (H) accounted for 37.87%, β-turns (E) accounted for 14.44%, and other structures (L) accounted for the remaining 47.68%. Random coils and α-helices were distributed uniformly throughout the protein; however, the α-helices were more obvious at the C terminal.

The HsCypD had four tetratricopeptide repeat (TPR) domains that contained TPR-1 and TPR-11, each having two hits. The final specific hit was for a cyclophilin_ABH_like, cyclophilin A, B, and H-like cyclophilin-type peptidylprolyl cis-trans isomerase (PPIase) domain, representing an archetypal cytosolic cyclophilin similar to human cyclophilin A, B, and H.

The predicted three-dimensional structure was mainly composed of three α-helices, eight β-strands, some β-turns, and random coils. It was barrel-shaped, and the top and bottom were a combination of loops and three α-helices, which connected with both ends of the β-strands. The α-helices were more obvious in the C terminal (Supplementary Figure 3).

### Phylogenetic relationship of HsCypD to homologs of other species

**Figure 2:**
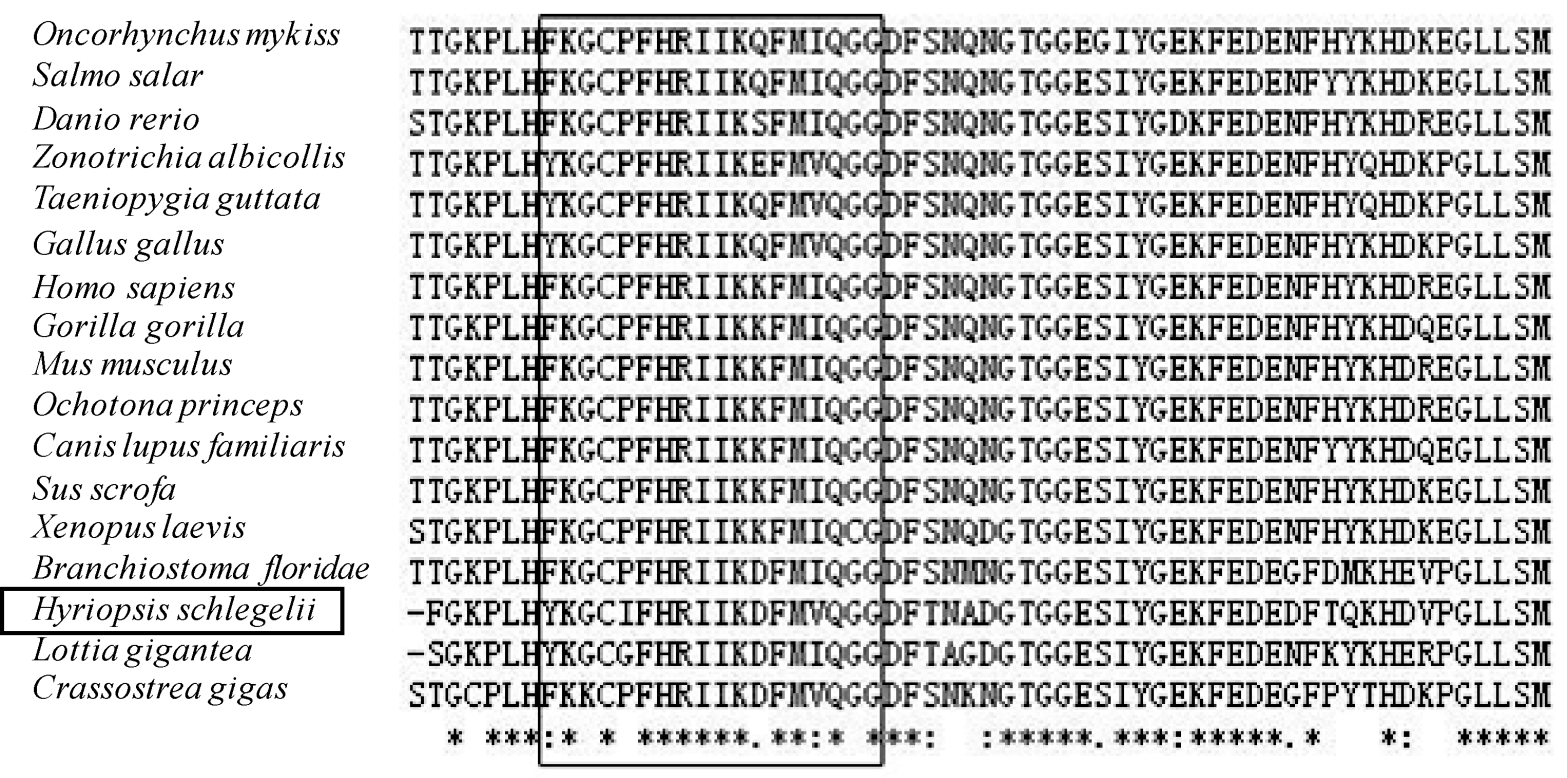
Identiﬁcation of a highly-conserved region across CypD amino acid sequences and their homologs

The HsCypD was homologous with CypD from other species (Supplementary Table 4), and exhibited the highest homology (61%) with *Lottia gigantea*. Multiple alignments revealed that the signature sequence of the CypD family could be identiﬁed in HsCypD (YKGCIFHRIIKDFMVQGG), and that the residues involved in CsA binding and PPIase activities were well conserved (Figure 2).

**Figure 3:**
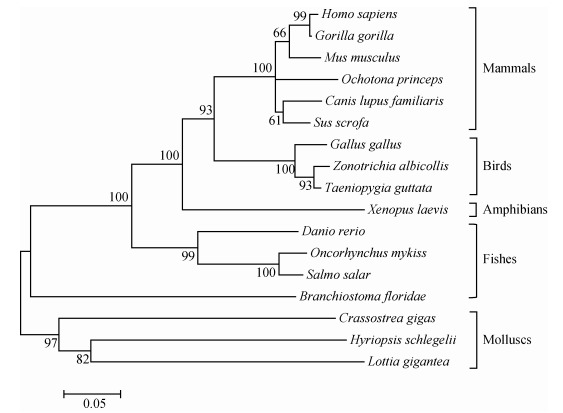
Phylogenetic tree (neighbor-joining) of CypD sequences including *Hyriopsis schlegelii* and 16 other species constructed using MEGA 4.0

Two major branches of vertebrates and invertebrates were classified on the CypD phylogenetic tree. HsCypD was found on the same branch as *Lottia gigantea*. *Homo sapiens* and *Gorilla gorilla* belonged to the same branch, and were the most distant from HsCypD (Figure 3). Furthermore, the phylogenetic tree based on CypD, CypC, and CypH sequences showed that HsCypH and CypDs from most species were grouped together. In particular, the CypC family and most of the CypD family were grouped together on a large branch. CypD from *Lottia gigantea* belonged to a lineage near the CypH group and CypH from *Oryctolagus cuniculus* belonged to a lineage near the CypC group (Supplementary Figure 4).

### Tissue expression proﬁle of HsCypD

**Figure 4:**
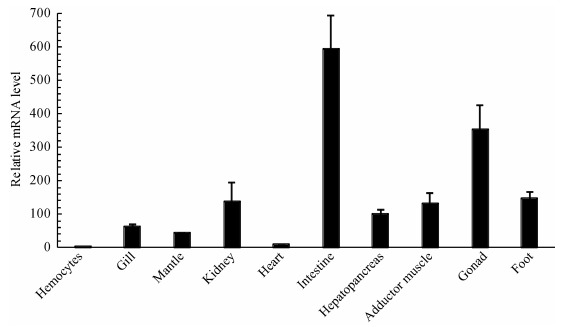
*HsCypD* mRNA expression levels in different tissues of *H. schlegelii*

The constitutive mRNA expression level of *HsCypD* was examined in 10 different tissues, including hemocytes, gill, mantle, kidney, heart, intestine, hepatopancreas, adductor muscle, gonad, and foot. The highest expression of *HsCypD* was detected in the intestine, with remarkable tissue expression patterns (Figure 4).

## DISCUSSION

HsCypD was identified and characterized in the freshwater pearl mussel *Hyriopsis schlegelii*. It comprised a cyclophilin-type peptidyl-prolyl cis-trans isomerase (PPIase) region, four significant TPR domains, and specific tertiary structure, as shared by other Cyps families (Blackburn et al., 2015; Ottiger et al., 1997; Wang et al., 2009). The PPIase region is highly-conserved in cyclophilins in vertebrates and invertebrates (Wang et al., 2009). The TPR domain connects to the single N-terminal cyclophilin domain of Cyp40 by a 30-amino acid linker (Taylor et al., 2001). Similar to the tertiary structure of human CypA and CypB (Mikol et al., 1994; Ottiger et al., 1997), HsCypD exhibited a right-handed barrel structure formed by eight β-strands. The top and bottom of this structure combined loops and three α-helices connected with both ends of the β-strands. HsCypD showed high conservativeness in these domains and tertiary structure.

The phylogenetic tree confirmed that HsCypD was more distantly related to CypDs from vertebrates than from invertebrates, similar to the evolutionary structure of CypA from *V. philippinarum* (Chen et al., 2011). Generally, the same types of cyclophilins (e.g., CypA and CypD) but isolated from different species are more closely related to each other than different types of cyclophilins from the same species (Lee et al., 2002), although there are exceptions (Chen et al., 2011). In this study, HsCypH belonged to a lineage near the CypD group on the phylogenetic tree, with CypD from *L. gigantea* grouped with CypH and CypH from *O. cuniculus* grouped with CypC (Supplementary Figure 4). These findings demonstrate the close phylogenetic relationship of cyclophilins and suggest that this family likely has a common origin and is highly conserved.

N-myristoylation is a lipid anchor modification of some proteins targeting them to membrane locations, thus transforming the function of the modified proteins, and plays a significant role in many cellular pathways, such as apoptosis, signal transduction, and alternative extracellular protein export (Borgese et al., 1996; Maurer-Stroh et al., 2002). PKC is an important neurotransmitter in intracellular signal transduction and participates in transmembrane signaling (Nishizuka, 1984). Another protein kinase, CKII, a highly conserved serine/threonine kinase of eukaryotic cells, is responsible for responding to growth factors (Marais et al., 1992). Tyrosine kinase is a key molecule in signal transduction and growth control (Cheng et al., 1993). The TPR domain can bind competitively to Hsp90 or Hsp70 and thus serve as co-chaperones (Young et al., 1998). The predicted HsCypD possessed these binding sites and domains. Thus, we speculated that HsCypD might have the ability to anchor to membranes, and might be involved in specific transfer processes of signal transduction and growth of cells, as well as performing as a chaperone.

Cyps are widely distributed in various tissues (Danielson et al., 1988; Qiu et al., 2009). The high expression of Cyps in tissues is related to certain functional mechanisms (Qiu et al., 2009; Watashi et al., 2005). In this study, the highest mRNA expression level of *HsCypD* was detected in the intestine. We speculated that HsCypD was very active in the intestine and might be involved in specific transfer processes of signal transduction and cytoprotection (Hausenloy et al., 2012; Tavecchio et al., 2013). Further systematic research is currently underway to characterize the functions and regulatory mechanisms of HsCypD.
